# Association of Circadian Rhythms With the Risk of Chronic Liver Disease: Findings From a Large Prospective Study

**DOI:** 10.14309/ctg.0000000000000949

**Published:** 2025-11-17

**Authors:** Rong Yang, Can Shen, Yu Jia, Yi Yao, Yiheng Zhou, Yu Cheng, Yonglang Cheng, Rui Zeng, Zhi Wan, Qian Zhao, Dongze Li, Xiaoyang Liao

**Affiliations:** 1 General Practice Ward/International Medical Center Ward, General Practice Medical Center, West China Hospital, Sichuan University, Chengdu, Sichuan, China;; 2 Department of Cardiology, West China Hospital, West China School of Medicine, Sichuan University, Chengdu, Sichuan, China;; 3 Department of Emergency Medicine, Disaster Medical Center, West China Hospital, West China School of Medicine, Sichuan University, Chengdu, Sichuan, China;; 4 Teaching & Research Section, General Practice Medical Center, West China Hospital, Sichuan University, Chengdu, Sichuan, China.

**Keywords:** circadian rhythms, chronic liver disease, relative amplitude, genetic susceptibility, UK biobank

## Abstract

**INTRODUCTION:**

The liver clock of hepatocytes is actively involved in regulating their proliferation, metabolism, oxidative stress response, and chronic liver disease (CLD) progression. However, the relationship between circadian rhythms and CLD remains poorly understood. This study aimed to examine the associations of circadian rhythms with metabolic dysfunction-associated steatotic liver disease, cirrhosis, and hepatocellular carcinoma.

**METHODS:**

This study included 94,006 participants from the UK Biobank. Circadian rhythms were assessed by a 7-day accelerometer by relative amplitude (RA), which indicates the difference between the most and least active periods. Cox regression and restricted cubic splines were used to evaluate the associations between circadian rhythms and CLD. Liver fat content and hepatic inflammation were additionally assessed using magnetic resonance imaging-measured proton density fat fraction and corrected T1 scores.

**RESULTS:**

During the follow-up of 9.8 years, individuals in the lowest quartile of RA had higher hazard ratios of 1.54 (95% CI: 1.32–1.78) for metabolic dysfunction-associated steatotic liver disease, 1.79 (95% CI: 1.38–2.32) for cirrhosis, and 1.65 (95% CI: 1.02–2.76) for hepatocellular carcinoma than those in the highest third quartile did. A dose‒response relationship between RA and CLD was observed (*P* < 0.001). Furthermore, there was a joint and independent relationship between polygenic risk scores, RA, and the CLD. RA was negatively correlated with proton density fat fraction and corrected T1 scores, demonstrating a dose‒response pattern (*P* < 0.001).

**DISCUSSION:**

Abnormal circadian rhythm is significantly associated with the risk of CLD, potentially due to increased liver fat content and hepatic inflammation. Therefore, disrupted circadian rhythms may be a risk factor for liver disease and represent a potential target for intervention.

## INTRODUCTION

Chronic liver diseases (CLDs) significantly contribute to global mortality, morbidity, diminished health-related quality of life, and economic burden (1). Among these diseases, metabolic dysfunction-associated steatotic liver disease (MASLD), which was recently redefined from nonalcoholic fatty liver disease, is the most prevalent form (2). This condition is characterized by the accumulation of excess fat within the liver in the absence of significant alcohol intake or other chronic liver diseases (3). With an estimated global prevalence of 32%, the prevalence of MASLD is expected to increase further due to rising rates of obesity and type 2 diabetes (3). It is a major driver of cirrhosis and hepatocellular carcinoma and accounts for most liver-related deaths (3,4), imposing a considerable health burden and substantial social and economic impacts (5,6). In light of the current scarcity of effective treatments for MASLD, prioritizing prevention is crucial, making the investigation of modifiable risk factors an urgent research priority (7).

Circadian rhythms, which are natural fluctuations in physiological and behavioral patterns, typically follow a 24-hour cycle. These rhythms are widespread throughout the natural world and are essential for maintaining health and internal balance (8,9). The central circadian pacemaker located in the suprachiasmatic nucleus of the hypothalamus is a key structure for regulating circadian rhythms (10). It receives light signals as synchronizing cues and communicates with peripheral clocks located in organs such as the liver through endocrine, neural, and metabolic signals. The circadian clock and liver metabolic homeostasis are interdependent, forming a foundational relationship that allows the body to efficiently use energy, synchronize tissue functions, and adapt to changes in internal and external environments (11).

In murine tissues, the liver expresses the greatest number of circadian rhythm-related genes, with studies identifying 536 genes exhibiting rhythmic expression patterns. In animal models, disruptions to circadian rhythms, whether direct or indirect due to physiological, metabolic, or molecular factors, are associated with the pathophysiology and progression of liver diseases, including MASLD, liver fibrosis, cirrhosis, and hepatocellular carcinoma. Notably, many proteins currently under investigation as drug targets for MASLD, such as sterol regulatory element-binding protein, acetyl-CoA carboxylase, peroxisome proliferator-activated receptors, and incretins, are regulated by circadian proteins (12,13). Therefore, disrupted circadian rhythms may serve as an important pathological basis for chronic liver diseases (13).

A previous cohort study revealed that disrupted diurnal rhythms of wrist temperature (lower amplitude) are associated with MASLD (14). Another study revealed that individuals engaged in shift work presented elevated liver fat content. However, to our knowledge, there have been no large-scale population studies reporting the relationship between circadian rhythms and liver diseases to date. Therefore, this study aimed to measure the circadian rhythms of a population using wrist-worn accelerometers over 7 days to prospectively analyze their relationships with MASLD, cirrhosis, hepatocellular carcinoma, and liver-related mortality and to cross-sectionally investigate the associations between circadian rhythms and liver fat content as well as hepatic fibroinflammation measured by MRI. In addition, this study explores whether these associations are linear.

## METHODS

### Study design and population

The Data set used in this study was obtained from the UK Biobank, a large-scale, prospective cohort involving more than half a million volunteers aged 37–63 years. The participants voluntarily consented to the study by providing written approval through online questionnaires for the use of their personal data. The research included detailed wrist-worn accelerometer data and MRI scans, along with long-term follow-up data, resulting in an extensive and valuable Data set. In line with rigorous ethical guidelines, the UK Biobank has been approved by the Northwest Multicenter Research Ethics Committee. Permission to access and use this Data set was also obtained from the Human Ethics Committee at West China Hospital, Sichuan University.

**Figure 1.**
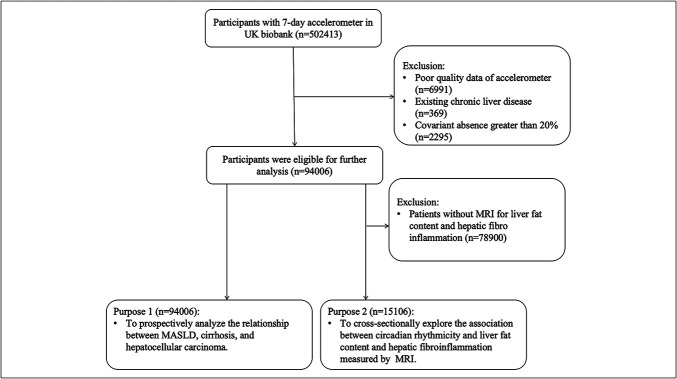
Flow diagram of the participants. MASLD, metabolic dysfunction-associated steatotic liver disease.

A total of 502,413 participants completed a 7-day accelerometer assessment. We excluded 6,991 participants due to poor-quality accelerometer data, 369 participants with pre-existing chronic liver disease (Table S1, http://links.lww.com/CTG/B419), and 2,295 participants with more than 20% missing covariate data. Poor-quality data were defined as participants with insufficient wear time, defined as a wear time <72 hours or no wear data for each hour within a 24-hour period, or devices identified by the UK Biobank as poorly calibrated. These exclusions were made to ensure the accuracy and reliability of the core exposure variable measurement (15). As a result, 94,006 participants were included in the final analysis. Moreover, for further analysis of circadian rhythms, liver fat content and hepatic fibroinflammation measured by MRI, after excluding 78,900 participants, 15,106 participants were included in a subcohort (Figure 1).

### Wrist-worn accelerometer

Between 2013 and 2015, more than 100,000 individuals wore an Axivity AX3 triaxial accelerometer on their dominant wrist for 1 week. The devices collected data at a frequency of 100 Hz across a dynamic range of ±8 g. The raw acceleration data were calibrated to account for gravity to increase accuracy. Nonwear periods were identified as consecutive hours of inactivity, characterized by a SD below 13.0 m across all 3 accelerometer axes.

For data analysis, nonparametric methods were applied to 5-second intervals of physical activity intensity data (UK Biobank field ID 90004) to calculate the relative amplitude (RA): This parameter quantifies the relative difference between most active 10-hour average (M10) and Least active 5-hour average (L5) over a 24-hour period. It is calculated as RA = (M10 - L5)/(M10 + L5). A higher RA indicates a more pronounced difference between the most and least active periods, and abnormal circadian rhythms were defined as the lowest quartile (Q1) of RA. Other circadian rhythm parameters were also calculated as described in a previous study (16): (i) L5: This parameter measures the average activity level during the least active 5-hour period of the day, typically associated with rest. (ii) M10: This represents the average activity level during the most active 10-hour period, reflecting the intensity of activity while awake. (iii) L5 onset time: This indicates the timing of the least active period, likely corresponding to sleep. (iv) M10 onset time: This is when the most active period begins, indicating early or late activity patterns. (v) Intradaily variability: This measures the fragmentation of the rest–activity rhythm, with higher values suggesting greater circadian disruption. Further details on the definition and validation of these parameters can be found in other published works (15).

### Genetic analyses

Genotyping procedures and imputation processes in the UK Biobank were performed using data from the UK BiLEVE and UK Biobank Axiom arrays. Further details can be found in other published sources. We selected single nucleotide polymorphisms (SNPs) associated with hepatocellular carcinoma and cirrhosis from recent genome-wide association studies (17), including specific variants such as PNPLA3 rs738409, HSD17B13 rs72613567, TM6SF2 rs58542926, MBOAT7 rs641738, and GCKR rs1260326. Each SNP was categorized with codes of 2 for homozygous presence, 1 for heterozygous presence, and 0 for absence of the variant. The polygenic risk scores (PRS) were computed using the following formula: (β1 × SNP1+ β2 × SNP2 + … + βn × SNPn) × (total number of SNPs/sum of the β-coefficients), where the β values were obtained from the original genome-wide association studies. Genetic risk was categorized into low risk for the lowest quartile of PRS scores, intermediate risk for the middle 2 quartiles, and high risk for the highest quartile.

### Outcome

The study results were determined by integrating National Health Service records, including both hospital admission data and mortality records. Access to hospital admission data extended until February 31, 2024, whereas mortality data were available until December 19, 2022. The study's follow-up period ended on these dates. The primary end point of interest was MASLD, which includes both hospitalization and mortality. This outcome was identified through hospital admission records, classified as either primary or secondary diagnoses (UKB data-field 41270), and death records, which capture both underlying and contributing causes (UKB data fields 40001 and 40002). MASLD was classified by the International Classification of Diseases, 10th revision (*ICD-10*), with specific codes K76.0 for fatty liver and K75.8 for other specified inflammatory liver diseases. The secondary outcomes included additional liver-related adverse events, such as cirrhosis (*ICD-10* codes K70.2, K70.3, K70.4, K74.0, K74.1, K74.2, K74.6, K76.6, and I85), liver cancer (*ICD-10* code C22.0), and liver-related mortality (*ICD-10* codes K70–K77, I85, or C22.0). Because only 35 cases of liver cancer and 34 cases of liver-related deaths were recorded, we combined the 2 events into 1. Table S2 (http://links.lww.com/CTG/B419) provides a comprehensive list of the *ICD-10* codes used for defining these outcomes.

The liver proton density fat fraction (PDFF) and iron-corrected T1 (cT1) score, assessed using MRI scans, served as additional outcome measures. The MRI and analysis protocols have been previously detailed, with participants scanned using a Siemens 1.5 T MAGNETOM Aera scanner and a 6-minute dual-echo Dixon Vibe protocol (18). This procedure yielded a water and fat-separated volumetric Data set from the neck to the knee. The average liver PDFF was determined from 9 regions of interest and carefully selected to exclude inhomogeneities, major vessels, and bile ducts. A cardiac-gated shortened modified look-locker inversion sequence was used to measure liver T1 values, and it can be adjusted to account for the influence of iron, resulting in a cT1 score, which is expressed in milliseconds and serves as an indirect indicator of hepatic fibroinflammatory activity. This metric, derived from MRI, has been confirmed for accuracy by comparison with liver histology and has shown practical use in clinical settings (19–22). According to previous studies, a cT1 score ≥780 ms indicates abnormal liver tissue, reflecting fibrosis and/or inflammatory activity (21).

### Covariate assessment

This study considered a wide range of socioeconomic factors, medical history, medication history, laboratory test results, and lifestyle factors to account for potential confounding influences. Those included age, which was calculated from the date of birth to the baseline assessment visit; body mass index (BMI), which was calculated from height and weight; and self-reported demographic details such as sex, ethnicity, and education level, with social deprivation quantified by the Townsend deprivation index (TDI). The TDI consists of 4 core indicators—unemployment, non-car ownership, non-home ownership, and household overcrowding. Its strength lies in its ability to capture area-level material deprivation through these 4 objective and easily obtainable census-based measures, rather than relying on abstract constructs such as access to healthy foods or job satisfaction. This index reflects the overall scarcity of material resources within a community, which systematically shapes the opportunities and choices available to individuals living in that area, thereby inherently encompassing aspects of residential environment, occupational prospects, and dietary quality (23). The medical history included hypertension and diabetes; the medication history focused on aspirin use; and the laboratory test indicators included blood glucose, glycated hemoglobin, blood lipids, albumin, and C-reactive protein. Lifestyle factors included self-reported smoking status (never, former, or current), alcohol consumption (calculated from the total units of average weekly intake across various beverages), and physical activity (metabolic equivalents hours per day for all physical activity). This comprehensive approach allowed us to adjust for various influences, providing a robust framework for understanding the relationships between the variables of interest and the outcomes being studied.

### Statistical analysis

Categorical variables are presented as n (%), and continuous variables are presented as the means ± SDs and medians (25th–75th). For the prospective analysis, we used a Cox proportional hazards model to investigate the association between RA and chronic liver disease. Hazard ratios (HRs) and 95% confidence intervals (CIs) for chronic liver disease were reported, with the lowest quartile used as the reference. To examine the potential dose‒response relationships between the intake of RA and the other 5 circadian rhythm parameters and the risk of chronic liver disease. We applied restricted cubic splines with 3 knots in fully adjusted models. Moreover, in the cross-sectional analysis, we used linear regression to assess the link of the RA with the arithmetic mean difference in the PDFF and cT1 scores. Similarly, restricted cubic splines for linear regression were also constructed. In these models, the relevant covariates were fully and consistently adjusted.

To determine whether genetic susceptibility (low vs moderate vs high) influences the relationship between RA and liver-related outcomes, we incorporated an interaction term into our regression analysis. The HR associated with this product term served as the interaction measure. Moreover, subgroup analyses were performed to investigate how RA is related to chronic liver disease, differentiated by factors such as age (<60 vs ≥60 years), sex (male vs female), and BMI (<25.0 vs 25.0–30.0 vs >30.0 kg/m^2^).

To ensure the reliability of our results, we conducted multiple sensitivity analyses. Initially, we excluded patients with MASLD, cirrhosis, and hepatocellular carcinoma with a follow-up period of less than 2 years. We subsequently considered nonliver-related mortality as a competing risk and additionally adjusted for aspirin use, genetic risk, and both simultaneously to account for potential confounding factors. After that, we excluded participants who exceeded baseline alcohol consumption limits (over 30 g per day for men and over 20 g per day for women). Finally, we conducted an analysis excluding nonwhite participants.

Statistical significance was defined as a 2-tailed *P* value < 0.05. Statistical analyses were performed using SPSS version 26.0 and R software version 4.3.0. Additional details on the statistical methods can be found in the supplementary materials (http://links.lww.com/CTG/B419).

## RESULTS

### Baseline characteristics

A total of 94,006 participants, with a mean age of 56.14 years, including 41,253 men (43.9%), were included in the prospective analysis. Compared with participants with normal circadian rhythms (RA, Q2–4), those with abnormal circadian rhythms (Q1 of RA) were more likely to be older; be male, nonwhite, and smokers; have a higher BMI; have lower levels of physical activity; and have a higher prevalence of comorbid hypertension and diabetes (Table 1).

**Table 1.** Basic information of the prospective analysis

Characteristics	Total	Normal circadian rhythmicity (Q2-4 of relative amplitude)	Abnormal circadian rhythmicity (Q1 of relative amplitude)	*P* value
Sample size, n (%)	94,005 (100%)	23,501 (25.0%)	70,504 (75.0%)	
Male, n (%)	41,253 (43.9%)	12,334 (52.5%)	28,919 (41.0%)	<0.001
Age (yr)	56.14 (7.82)	56.74 (7.96)	55.95 (7.76)	<0.001
White, n (%)	91,905 (97.8%)	22,626 (96.3%)	69,279 (98.3%)	<0.001
Townsend deprivation Index	−1.73 (2.81)	−1.20 (3.08)	−1.91 (2.70)	<0.001
Season				<0.001
Spring	20,185 (21.5%)	5,214 (22.2%)	14,971 (21.2%)	
Summer	22,069 (23.5%)	5,125 (21.8%)	16,944 (24.0%)	
Autumn	25,032 (26.6%)	6,103 (26.0%)	18,929 (26.8%)	
Winter	26,719 (28.4%)	7,059 (30.0%)	19,660 (27.9%)	
Education				<0.001
College or University degree	40,843 (43.4%)	10,020 (42.6%)	30,823 (43.7%)	
A AS level or equivalent	12,381 (13.2%)	3,029 (12.9%)	9,352 (13.3%)	
O levels or equivalent	22,912 (24.4%)	5,518 (23.5%)	17,394 (24.7%)	
Other	17,869 (19.0%)	4,934 (21.0%)	12,935 (18.3%)	
Alcohol consumption (g/d)	10.72 (10.51)	11.27 (12.03)	10.53 (9.95)	<0.001
Smoking status, n (%)				<0.001
Never	53,636 (57.1%)	12,281 (52.3%)	41,355 (58.7%)	
Former	33,890 (36.1%)	8,745 (37.2%)	25,145 (35.7%)	
Current	6,479 (6.9%)	2,475 (10.5%)	4,004 (5.7%)	
BMI (kg/m^2^)	26.69 (4.50)	28.49 (5.27)	26.09 (4.05)	<0.001
Physical activity (MET hr/wk)	42.21 (40.66)	36.35 (38.18)	44.17 (41.27)	<0.001
Hypertension, n (%)	39,891 (42.4%)	11,029 (46.9%)	28,862 (40.9%)	<0.001
Diabetes, n (%)	3,206 (3.4%)	1,593 (6.8%)	1,613 (2.3%)	<0.001
HbAIC (%)	35.38 (5.55)	36.44 (7.20)	35.03 (4.83)	<0.001
Triglyceride (mmol/L)	1.66 (0.97)	1.84 (1.06)	1.60 (0.93)	<0.001
Cholesterol (mmol/L)	5.73 (1.11)	5.60 (1.16)	5.77 (1.09)	<0.001
Albumin (g/L)	45.36 (2.58)	45.14 (2.66)	45.43 (2.54)	<0.001
HDL-c (mmol/L)	1.49 (0.38)	1.39 (0.37)	1.52 (0.38)	<0.001
LDL-c (mmol/L)	3.57 (0.85)	3.52 (0.88)	3.59 (0.84)	<0.001
C-reactive protein (mg/L)	2.24 (3.90)	2.86 (4.58)	2.03 (3.63)	<0.001

BMI, body mass index; HbAIC, glycated hemoglobin; HDL-c, high-density lipoprotein cholesterol; LDL-c, low-density lipoprotein cholesterol; MET, metabolic equivalents.

### Association of circadian rhythms with chronic liver disease events

During a median follow-up of 9.8 years (range: 9.2–10.3 years), 814 participants (0.8%) were diagnosed with MASLD, 265 (0.2%) with cirrhosis, and 69 (0.1%) with hepatocellular carcinoma. After adjusting for potential confounders, individuals with abnormal circadian rhythms presented HRs of 1.54 (95% CI: 1.32–1.78) for MASLD, 1.79 (95% CI: 1.38–2.32) for cirrhosis, and 1.65 (95% CI: 1.02–2.76) for hepatocellular carcinoma (Table 2). The cumulative event rate was higher (Figure S1, http://links.lww.com/CTG/B419).

**Table 2.** Cox proportional hazards regression analysis of relative amplitude with MASLD, cirrhosis, and hepatocellular carcinoma

	Model 1		Model 2		Model 3	
	HR (95% Cl)	*P*	HR (95% Cl)	*P*	HR (95% Cl)	*P*
MASLD						
Q1 of relative amplitude	2.48 (2.16, 2.85)	<0.001	2.44 (2.13, 2.81)	<0.001	1.54 (1.32, 1.78)	<0.001
Q2-4 of relative amplitude	Reference		Reference		Reference	
Cirrhosis						
Q1 of relative amplitude	3.00 (2.36, 3.82)	<0.001	2.73 (2.14, 3.47)	<0.001	1.79 (1.38, 2.32)	<0.001
Q2-4 of relative amplitude	Reference		Reference		Reference	
Hepatocellular carcinoma						
Q1 of relative amplitude	2.97 (1.86, 4.77)	<0.001	2.63 (1.64, 4.24)	<0.001	1.65 (1.02, 2.76)	0.049
Q2-4 of relative amplitude	Reference		Reference		Reference	

Model 1: Without adjustment for confounding factors. Model 2: Additional adjustment for sex and age. Model 3: Additional adjustment for race, Townsend deprivation index, season, education, alcohol consumption, smoking status, body mass index, physical activity, hypertension, diabetes, triglycerides and cholesterol.

MASLD, metabolic dysfunction-associated steatotic liver disease.

**Figure 2.**
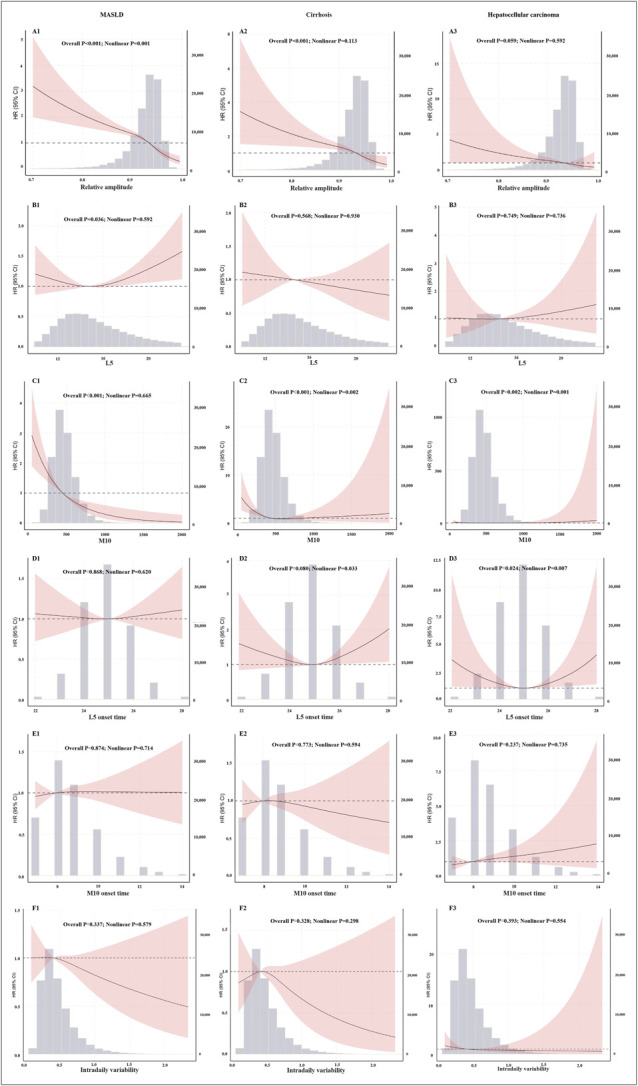
Restricted cubic splines analysis of circadian rhythms with chronic liver disease. L5: Least active 5-hour average; M10: Most active 10-hour average; A(1)-F(1): Restricted cubic splines analysis of circadian rhythms with MASLD; A(2)-F(2): Restricted cubic splines analysis of circadian rhythms with cirrhosis; A(3)-F(3): Restricted cubic splines analysis of circadian rhythms with hepatocellular carcinoma. HR, hazard ratio; MASLD, metabolic dysfunction-associated steatotic liver disease.

The results revealed a significant dose‒response relationship between RA and CLD (*P* < 0.001). Specifically, the relationships between RA and both MASLD and cirrhosis were nonlinear: as RA increased, the risks of MASLD and cirrhosis decreased, ultimately becoming protective. Furthermore, M10 was associated with all listed CLDs, L5 was associated with MASLD, and L5 onset time was associated with hepatocellular carcinoma (Figure 2).

### Modification effect of genetic susceptibility

**Figure 3.**
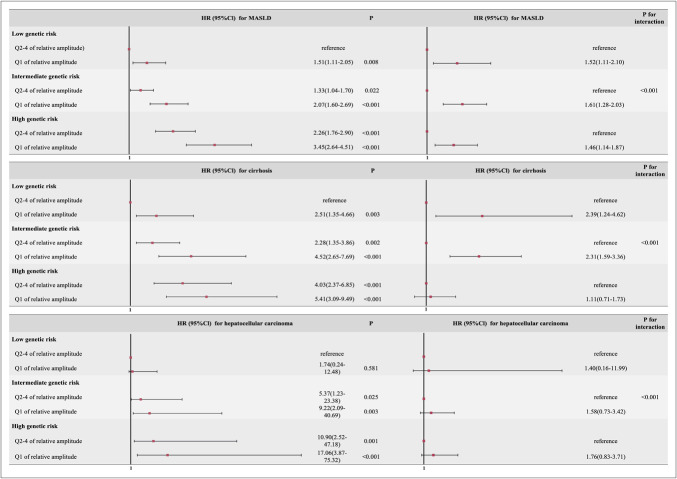
Independent and joint relationships between polygenic risk scores, relative amplitude, and chronic liver disease. HR, hazard ratio.

This study identified a joint and independent relationship between PRS, RA, and the CLD. Compared with the other groups, the group with high genetic risk and abnormal circadian rhythms presented significantly greater risks of MASLD, cirrhosis, and hepatocellular carcinoma, with HRs of 3.45 (95% CI: 2.64–4.41), 5.41 (95% CI: 3.09–9.49), and 17.06 (95% CI: 3.87–75.32), respectively (Figure 3).

The risks associated with carrying the genetic variants of PNPLA3, HSD17B13, TM6SF2, MBOAT7, and GCKR. The results indicated that all genes significantly modified the relationship between RA and MASLD. In addition, MBOAT7 and GCKR significantly modified the relationship between RA and cirrhosis, and GCKR significantly modified the relationship between RA and hepatocellular carcinoma (Tables S3–S7, http://links.lww.com/CTG/B419).

### Subgroup and sensitivity analyses

In the subgroup analysis, sex, age, and BMI did not affect the effect of RA on the risk of these outcomes (Tables S8–S10, http://links.lww.com/CTG/B419). According to the sensitivity analysis, the risks of MASLD, cirrhosis, and hepatocellular carcinoma remained elevated in the abnormal circadian rhythm group (Table S11, http://links.lww.com/CTG/B419).

### Circadian rhythms, quantitative liver fat content, and fibroinflammatory activity

After excluding individuals who did not undergo MRI, a total of 15,106 participants were included in the cross-sectional analysis, with a mean age of 55.30 years, of whom 11,835 were men (78.3%) (Table S12, http://links.lww.com/CTG/B419).

**Figure 4.**
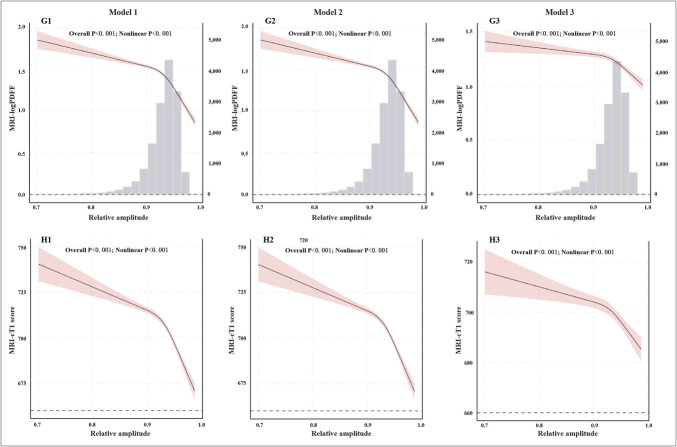
Restricted cubic splines analysis of the relative amplitude with logPDFF and cT1 scores. Model 1: Without adjustment for confounding factors; Model 2: Additional adjustment for sex and age; Model 3: Additional adjustment for race, town-level deprivation index, season, education, alcohol consumption, smoking status, body mass index, physical activity, hypertension, diabetes, triglycerides and cholesterol. cT1, corrected T1; PDFF, proton density fat fraction.

The cross-sectional analysis revealed a dose‒response relationship between RA and both PDFF and cT1 scores (*P* < 0.001), exhibiting a nonlinear pattern (*P* < 0.001). As RA increased, the PDFF gradually decreased, with a marked decline observed beyond an RA level of 0.9. The association with cT1 scores was initially minimal but progressively decreased once the RA exceeded 0.9 (Figure 4).

Linear regression analysis revealed that the abnormal circadian rhythm group had a significantly greater PDFF by 0.10 (95% CI: 0.07, 0.12) and cT1 score by 8.35 (95% CI: 6.15, 10.56) than did the high RA group, with both differences being statistically significant (*P* < 0.001) (Table S13, http://links.lww.com/CTG/B419).

## DISCUSSION

This study investigated the association between circadian rhythm disorders and liver disease using a large-scale population sample. The cohort study findings revealed that individuals with circadian rhythm disorders had an increased risk of CLD compared with those with normal circadian rhythms. In addition, a dose‒response relationship was observed between circadian rhythm disruption and an increased risk of liver-related diseases. The study also demonstrated that genetic factors modify the relationship between circadian rhythms and CLD. Further analysis indicated that abnormal circadian rhythms were negatively correlated with liver fat content and hepatic fibro-inflammation. This study underscores the importance of circadian rhythm regulation for liver health and offers valuable insights into other health issues linked to circadian rhythm disturbances.

Numerous previous studies have investigated the mechanisms by which circadian rhythm impacts liver disease. These include the effects of circadian rhythm disruption on fatty acid synthesis and breakdown, which increase the risk of fatty liver and metabolic syndrome, as well as how the amplitude of the liver's biological clock affects the expression and activity of drug-metabolizing enzymes (13,24). A reduced amplitude potentially leads to decreased drug metabolism and increased risk of drug toxicity (25). In a recent cohort study of 300,000 participants without liver disease at baseline, inappropriate sleep patterns (sleeping >8 hours or <7 hours per day), a nocturnal chronotype, frequent insomnia, snoring, and habitual daytime drowsiness were associated with a 16–78% increased risk of developing severe MASLD (26). These findings align with our study results. Further analysis revealed a significant dose‒response relationship between circadian rhythms and the risks of MASLD, cirrhosis, and hepatocellular carcinoma, suggesting that as RA increased, the risks of these conditions gradually decreased, eventually transitioning into protective factors. Notably, the relationships with MASLD and cirrhosis were nonlinear, whereas the association with hepatocellular carcinoma was linear. These findings suggest that the nonlinear relationship between MASLD and cirrhosis may reflect a more pronounced influence of the circadian rhythm on the early stages of metabolic liver disease, whereas the linear relationship with hepatocellular carcinoma implies a sustained protective role of the circadian rhythm in reducing hepatocellular carcinoma risk. From an intervention perspective, maintaining healthy circadian rhythms through lifestyle interventions—such as regular schedules, controlled light exposure, and a balanced diet—may help prevent fatty liver and cirrhosis. Such interventions could stabilize cellular metabolism, reduce inflammation, and enhance antioxidant defenses (27,28).

This study analyzed the effects of this modification on genetic susceptibility. Among individuals with greater genetic risk, those in the abnormal circadian rhythm group had a significantly greater risk of developing chronic liver disease; however, the risks were not simply additive. Recent studies have revealed that more than 80% of rhythmic gene expression and three-dimensional chromatin interactions in the liver are jointly regulated by genetic and nutritional factors (29). In individuals at greater genetic risk, certain SNPs, potentially located in enhancer or promoter regions of genes regulating lipid metabolism, inflammatory responses, or fibrotic pathways, may be present in liver cells. Under normal circadian rhythms, the effects of these genetic variants may remain minimal; however, when circadian rhythms are severely disrupted and the output signals of the core circadian clock become dysregulated, these negative effects may be amplified, leading to impaired toxin metabolism, excessive lipid accumulation, and increased release of proinflammatory cytokines. Through chromatin interaction analysis, researchers have demonstrated that estrogen-related receptor gamma regulates the temporal expression of key pathways, such as lipid metabolism, the urea cycle, and amino acid metabolism, by modulating dynamic enhancer‒promoter interactions. Genetic variants in individuals with high genetic risk that affect the expression or function of estrogen-related receptor gamma may predispose them to desynchronization of hepatic metabolic networks under circadian rhythm disruption, thereby substantially increasing the risk of metabolic disorders and liver injury (29). Similarly, single-gene analyses indicated that in carriers positive for TM6SF2, MBOAT7, and GCKR, the association between circadian rhythm disruption and fatty liver disease was attenuated, whereas in GCKR-positive individuals, the associations between circadian rhythm disruption and cirrhosis and hepatocellular carcinoma were enhanced. Previous studies have reported that the presence of these genetic variants is associated with increased risks of fatty liver, cirrhosis, and hepatocellular carcinoma, which contradicts the findings of this study (30–32). This discrepancy may be due to complex regulatory mechanisms between genetic variations and circadian rhythms. For example, genetic variations may mask the effects of circadian rhythms under certain conditions, or RA levels themselves may result from metabolic states such as inflammation and insulin sensitivity, whereas genetic variations primarily influence lipid metabolism pathways (33).In addition, the results revealed a nonlinear relationship between circadian rhythms and fatty liver as well as liver fat content. If genetic variations alter the metabolic mechanisms of circadian rhythms, this nonlinear relationship may lead to the attenuation or even reversal of the effects of circadian rhythms in individuals positive for these genes. Finally, the presence of unadjusted confounding factors may weaken the observed effects of circadian rhythms.

This study further used MRI to assess liver fat content and hepatic inflammation in the population. The analysis indicated that an abnormal circadian rhythm may be an independent risk factor of liver fat accumulation and hepatic inflammation. Increased liver fat content represents an early stage in the development of MASLD, whereas hepatic inflammation is a key mechanism in the onset and progression of chronic liver disease (34). Previous studies have also established strong associations between abnormal circadian rhythms and obesity, insulin resistance, and metabolic syndrome, all of which are critical drivers of liver fat accumulation and inflammation (33). Therefore, circadian rhythm disruption may facilitate the entire process from MASLD to hepatocellular carcinoma through these mechanisms, further confirming the direct impact of circadian rhythm disruptions on liver fat and inflammation and providing evidence linking circadian rhythm abnormalities from metabolic dysregulation to organ damage.

This study has several strengths. First, the large sample size and extended follow-up period provide a more timely and reliable risk assessment. In addition, the dose‒response relationships between RA and MASLD, cirrhosis, and hepatocellular carcinoma revealed detailed associations between circadian rhythm and liver disease, offering deeper insights into the nonlinear relationship between circadian rhythm levels and disease risk.

Regarding limitations, although our findings demonstrated a strong independent association between circadian rhythm disruption and CLD, this observational study cannot establish a definitive causal relationship. Nevertheless, several lines of evidence suggest that the relationship may be causal in nature. The prospective design ensured the temporal precedence of circadian rhythm disruption before CLD diagnosis. We observed a nonlinear dose–response relationship through restricted cubic spline analysis, indicating that the risk of CLD increased with greater circadian rhythm disruption. Most notably, the study revealed that genetic susceptibility variants in key hepatic metabolic pathways, including PNPLA3, HSD17B13, TM6SF2, MBOAT7, and GCKR, modified the observed associations. This study also has limitations related to incomplete adjustment for potential confounding factors such as lifestyle, occupation, and environmental exposures. To minimize bias, we adjusted for self-reported smoking status, alcohol consumption, and physical activity, as well as BMI and lipid profiles, which serve as proxy measures of long-term dietary patterns, and the TDI, a composite indicator of socioeconomic status closely related to living environment, occupation, and access to healthy foods. The results remained robust after these adjustments. On the other hand, the possibility of reverse causation cannot be completely ruled out. Therefore, although our findings support a potential causal relationship between circadian rhythm disruption and CLD, interventional studies are warranted to establish causality conclusively. In addition, although this study benefited from a large sample size and sufficient statistical power, the absolute incidence of MASLD, particularly hepatocellular carcinoma, was relatively low, which may limit the stability of the models in certain extreme subgroups. Future studies involving larger consortium-based collaborations or specialized cohorts focused on high-risk populations are needed to further validate our findings.

Circadian rhythm disruption is associated with an increased risk of CLD, and genetic susceptibility influences this association. Further analysis revealed that circadian rhythm disruption led to CLD through increased liver fat content and hepatic inflammation. Therefore, circadian rhythm disruption can serve as a risk factor of CLD and a potential target for intervention.

## CONFLICTS OF INTEREST

**Guarantor of the article:** Xiaoyang Liao, MD.

**Specific author contributions:** R.Y.: Conceptualization, Methodology, Writing—Original draft preparation, Writing—Reviewing and Editing; C.S.: Methodology, Writing—Original draft preparation, Writing—Reviewing and Editing, Visualization; Y.J.: Writing—Reviewing and Editing, Data Curation; Y.Y.: Writing—Reviewing and Editing, Data Curation; Y.Z.: Writing—Reviewing and Editing, Data Curation; Y.C.: Writing—Reviewing and Editing, Data Curation; Y.C.: Writing—Reviewing and Editing; R.Z.: Writing—Reviewing and Editing; Z.W.: Writing—Reviewing and Editing; Q.Z.: Writing—Reviewing and Editing; D.L.: Conceptualization, Supervision; X.L.: Conceptualization, Supervision. All the authors approved the final draft of the manuscript.

**Financial support:** This work was supported financially by grants from “Active Health and Technological Response to Population Aging” Key Special Project (No.2024YFC3607700), Sichuan Science and Technology Program (No. 2024NSFSC0661, 2024NSFSC1534, 24ZDYF0065), 135 Project for Disciplines of Excellence-Clinical Research Incubation Project (No. 2023HXFH002) and Postdoctor Research Fund of West China Hospital, Sichuan University (No. 2024HXBH067).

**Potential competing interests:** The authors declare that they have no conflict of interest.

**Availability of data and materials:** Data and materials can be obtained at https://ukbiobank.dnanexus.com/panx/projects

**IRB approval statement:** Ethics approval for the UK Biobank study was given by the NHS National Research Ethics Service (16/NW/0274). The experimental protocols were established according to the ethical guidelines of the Helsinki Declaration. Written informed consent was obtained from individual or guardian participants. All methods were carried out in accordance with guidelines and regulations developed by the UK Biobank. Data usage was approved by the Human Ethical Committee of the West China Hospital of Sichuan University (2023–1207).

**Study Highlights**

**WHAT IS KNOWN**

✓ Hepatocyte circadian clocks regulate liver metabolism and disease progression
✓ Population-level evidence linking objective circadian metrics to chronic liver disease remains limited.

**WHAT IS NEW HERE**

✓ Establishes circadian disruption as an independent and modifiable risk factor of chronic liver disease.
✓ Links relative amplitude to hepatic fat accumulation and inflammation, suggesting circadian rhythms may mitigate metabolic stress.

## Supplementary Material

## ABBREVIATIONS:

BMI: body mass index
CI: confidence interval
CLD: chronic liver disease
cT1: iron-corrected T1
HR: hazard ratio
L5: least active 5-hour average
M10: most active 10-hour average
MASLD: metabolic dysfunction-associated steatotic liver disease
PDFF: proton density fat fraction
PRS: polygenic risk score
RA: relative amplitude
SNP: single-nucleotide polymorphism
TDI: Townsend deprivation index
