# Supplementary material for: Association of Circadian Rhythms With the Risk of Chronic Liver Disease: Findings From a Large Prospective Study
Source: Clin Transl Gastroenterol. 2025 Nov 17;17(1):e00949. doi: 10.14309/ctg.0000000000000949 (PMC12818853; doi:10.14309/ctg.0000000000000949)
Supplement: Supplementary file 1 [file ct9-17-e00949-s001.pdf]

Table S1. Definitions and codes used for defining existing liver disease at baseline.

Liver Disease at Baseline	Description	ICD10	Data Field	Assessment Centre Data
Liver Disease	Any alcoholic liver disease	K70	41270	International Classification of Diseases and related health problems
	Any toxic liver disease	K71		
	Hepatic failure, not elsewhere classified	K72		
	Chronic hepatitis, not elsewhere classified	K73		
	Fibrosis and cirrhosis of liver	K74		
	Other inflammatory liver diseases	K75		
	Other diseases of liver	K76		
	Liver failure/ cirrhosis	/	20002	Verbal interview
	Infective/viral hepatitis	/		
	Non-infective hepatitis	/		
	Hepatitis	/		

Table S2. Definitions and codes used for defining chronic liver disease.

Outcome		Description	ICD10	Assessment Centre Data
Chronic Liver Disease (Data field 94006)	Metabolic dysfunction－associated steatotic liver disease	Fatty (change of) liver, not elsewhere classified	K76.0	International Classification of Diseases and related health problems
		Other specified inflammatory liver diseases	K75.8	
	Liver cirrhosis	Alcoholic fibrosis and sclerosis of liver	K70.2	
		Alcoholic cirrhosis of liver	K70.3	
		Alcoholic hepatic failure	K70.4	
		Hepatic fibrosis	K74.0	
		Hepatic sclerosis	K74.1	
		Hepatic fibrosis with hepatic sclerosis	K74.2	
		Other and unspecified cirrhosis of liver	K74.6	
		Portal hypertension	K76.6	
		Oesophageal varices	I85	
	Liver cancer	Liver cell carcinoma	C22.0	
	Liver-related mortality	Any alcoholic liver disease	K70	
		Any toxic liver disease	K71	
		Hepatic failure, not elsewhere classified	K72	
		Chronic hepatitis, not elsewhere classified	K73	
		Fibrosis and cirrhosis of liver	K74	
		Other inflammatory liver diseases	K75	
		Other diseases of liver	K76	
		Liver disorders in diseases classified elsewhere	K77	
		Oesophageal varices	I85	
		Liver cell carcinoma	C22.0	

Table S3. Modification effect of PNPLA3 on the association between relative amplitude and CLD.

	PNPLA3 (-)		PNPLA3 (+)		HR (95%Cl)	P for interaction
	HR (95%Cl)	P	HR (95%Cl)	P		
MASLD						
Q1 of relative amplitude	1.49 (1.28, 1.74)	<0.001	1.79 (1.06, 3.04)	0.031	1.68 (1.16, 2.42)	0.006
Q2-4 of relative amplitude	reference		reference		reference	
Cirrhosis						
Q1 of relative amplitude	1.86 (1.42, 2.44)	<0.001	1.03 (0.39, 2.67)	0.957	1.22 (0.60, 2.48)	0.858
Q2-4 of relative amplitude	reference		reference		reference	
Hepatocellular carcinoma						
Q1 of relative amplitude	1.98 (1.14, 3.43)	0.015	0.32 (0.05, 1.99)	0.221	1.06 (0.25, 4.40)	0.940
Q2-4 of relative amplitude	reference		reference		reference	

Table S4. Modification effect of HSD17B13 on the association between relative amplitude and CLD.

	HSD17B13 (-)		HSD17B13(+)		HR (95%Cl)	P for interaction
	HR (95%Cl)	P	HR (95%Cl)	P		
MASLD						
Q1 of relative amplitude	1.52 (1.30, 1.77)	<0.001	1.54 (0.99, 2.39)	0.056	1.87 (1.37, 2.55)	<0.001
Q2-4 of relative amplitude	reference		reference		reference	
Cirrhosis						
Q1 of relative amplitude	2.00 (1.52, 2.63)	<0.001	0.61 (0.26, 1.42)	0.250	1.31 (0.69, 2.46)	0.412
Q2-4 of relative amplitude	reference		reference		reference	
Hepatocellular carcinoma						
Q1 of relative amplitude	1.81 (1.02, 3.20)	0.042	1.23 (0.35, 4.30)	0.741	2.07 (0.75, 5.70)	0.162
Q2-4 of relative amplitude	reference		reference		reference	

Table S5. Modification effect of TM6SF2 on the association between relative amplitude and the CLD.

	TM6SF2 (-)		TM6SF2 (+)		HR (95%Cl)	P for interaction
	HR (95%Cl)	P	HR (95%Cl)	P		
MASLD						
Q1 of relative amplitude	1.58 (1.36,1.85)	<0.001	0.99 (0.60,1.64)	0.979	1.46 (1.01,2.17)	0.047
Q2-4 of relative amplitude	reference		reference		reference	
Cirrhosis						
Q1 of relative amplitude	1.90 (1.45,2.49)	<0.001	0.92 (0.38,2.23)	0.851	1.32 (0.68,2.57)	0.417
Q2-4 of relative amplitude	reference		reference		reference	
Hepatocellular carcinoma						
Q1 of relative amplitude	1.60 (0.95,2.69)	0.078	2.25 (0.17,30.31)	0.541	1.05 (0.25,4.30)	0.951
Q2-4 of relative amplitude	reference		reference		reference	

Table S6. Modification effect of MB0AT7 on the association between relative amplitude and the CLD.

	MB0AT7 (-)		MB0AT7 (+)		HR (95%Cl)	P for interaction
	HR (95%Cl)	P	HR (95%Cl)	P		
MASLD						
Q1 of relative amplitude	1.56 (1.33,1.82)	<0.001	1.32 (0.87,2.00)	0.194	1.94 (1.42,2.65)	<0.001
Q2-4 of relative amplitude	reference		reference		reference	
Cirrhosis						
Q1 of relative amplitude	1.90 (1.44,2.50)	<0.001	1.11 (0.52,2.36)	0.784	1.78 (1.02,3.12)	0.044
Q2-4 of relative amplitude	reference		reference		reference	
Hepatocellular carcinoma						
Q1 of relative amplitude	1.67 (0.97,2.86)	0.065	1.25 (0.25,6.19)	0.789	0.85 (0.67,1.08)	0.177
Q2-4 of relative amplitude	reference		reference		reference	

Table S7. Modification effect of the GCKR on the association between the relative amplitude and CLD.

	GCKR (-)		GCKR (+)		HR (95%Cl)	P for interaction
	HR (95%Cl)	P	HR (95%Cl)	P		
MASLD						
Q1 of relative amplitude	1.53 (1.31,1.79)	<0.001	1.39 (0.88,2.19)	0.156	1.90 (1.38,2.62)	<0.001
Q2-4 of relative amplitude	reference		reference		reference	
Cirrhosis						
Q1 of relative amplitude	1.76 (1.34,2.32)	<0.001	1.82 (0.81,4.10)	0.150	2.19 (1.32,3.64)	0.003
Q2-4 of relative amplitude	reference		reference		reference	
Hepatocellular carcinoma						
Q1 of relative amplitude	1.39 (0.78,2.46)	0.264	3.50 (1.05,11.66)	0.042	4.18 (1.978,8.86)	<0.001
Q2-4 of relative amplitude	reference		reference		reference	

Table S8. Subgroup analysis of the association between relative amplitude and CLD by sex.

	Male		Female		HR (95%Cl)	P for interaction
	HR (95%Cl)	P	HR (95%Cl)	P		
MASLD						
Q1 of relative amplitude	1.62 (1.31,2.00)	<0.001	1.45 (1.17,1.79)	0.001	1.64 (0.62,4.35)	0.317
Q2-4 of relative amplitude	reference		reference		reference	
Cirrhosis						
Q1 of relative amplitude	1.68 (1.22,2.32)	0.002	1.97 (1.27,3.03)	0.002	1.43 (0.87,2.35)	0.161
Q2-4 of relative amplitude	reference		reference		reference	
Hepatocellular carcinoma						
Q1 of relative amplitude	1.37 (0.72,2.62)	0.338	2.19 (0.94,5.08)	0.068	1.64 (0.62,4.35)	0.317
Q2-4 of relative amplitude						

Table S9. Subgroup analysis of the associations between the relative amplitude with CLD by age.

	<60y		≥60y		HR (95%Cl)	P for interaction
	HR (95%Cl)	P	HR (95%Cl)	P		
MASLD						
Q1 of relative amplitude	1.48 (1.22,1.79)	<0.001	1.65 (1.31,2.09)	<0.001	1.01 (0.76,1.34)	0.950
Q2-4 of relative amplitude	reference		reference		reference	
Cirrhosis						
Q1 of relative amplitude	1.94 (1.35,2.78)	<0.001	1.69 (1.16,2.46)	0.007	0.86 (0.53,1.39)	0.539
Q2-4 of relative amplitude	reference		reference		reference	
Hepatocellular carcinoma						
Q1 of relative amplitude	2.60 (1.10,6.14)	0.030	1.33 (0.70,2.54)	0.390	0.56 (0.21,1.49)	0.243
Q2-4 of relative amplitude	reference		reference		reference	

Table S10. Subgroup analysis of the associations of relative amplitude with CLD by body mass index.

	<25.0 kg/m²		25.0-30.0 kg/m²		≥30.0 kg/m²		HR (95%Cl)	P for interaction
	HR (95%Cl)	P	HR (95%Cl)	P	HR (95%Cl)	P		
MASLD								
Q1 of relative amplitude	2.13 (1.40,3.22)	<0.001	1.51 (1.20,1.91)	0.001	1.56 (1.27,1.91)	<0.001	0.85 (0.70,1.04)	0.116
Q2-4 of relative amplitude	reference		reference		reference		reference	
Cirrhosis								
Q1 of relative amplitude	2.70 (1.53,4.76)	0.001	1.57 (1.03,2.39)	0.036	1.64 (1.12,2.40)	0.011	0.80 (0.57,1.12)	0.195
Q2-4 of relative amplitude	reference		reference		reference		reference	
Hepatocellular carcinoma								
Q1 of relative amplitude	0.95 (0.25,3.60)	0.938	1.86 (0.83,4.16)	0.132	1.65 (0.76,3.58)	0.202	1.21 (0.62,2.37)	0.576
Q2-4 of relative amplitude	reference		reference		reference		reference	

Table S11. Sensitivity analysis

	Model 4		Model 5		Model 6		Model 7		Model 8		Model 9		Model 10	
	**HR (95%CI)**	**P**	**HR (95%CI)**	**P**	**HR (95%CI)**	**P**	**HR (95%CI)**	**P**	**HR (95%CI)**	**P**	**HR (95%CI)**	**P**	**HR (95%CI)**	**P**
MASLD														
Q1 of relative amplitude	1.54 (1.33,1.80)	<0.001	1.49 (1.27,1.74)	<0.001	1.49 (1.27,1.75)	<0.001	1.54 (1.32,1.78)	<0.001	1.53 (1.32,1.78)	<0.001	1.53 (1.32,1.78)	<0.001	1.51 (1.30,1.76)	<0.001
Q2-4 of relative amplitude	reference		reference		reference		reference		reference		reference		reference	
Cirrhosis														
Q1 of relative amplitude	1.81 (1.39,2.35)	<0.001	1.61 (1.20,2.16)	0.001	1.66 (1.26,2.20)	<0.001	1.78 (1.37,2.31)	<0.001	1.78 (1.37,2.31)	<0.001	1.77 (1.37,2.30)	<0.001	1.77 (1.36,2.31)	<0.001
Q2-4 of relative amplitude	reference		reference		reference		reference		reference		reference		reference	
Hepatocellular carcinoma														
Q1 of relative amplitude	1.65 (0.98,2.79)	0.059	1.38 (0.76,2.51)	0.287	1.57 (0.92,2.71)	0.104	1.61 (0.96,2.70)	0.069	1.63 (0.98,2.73)	0.062	1.57 (0.95,2.66)	0.080	-	-
Q2-4 of relative amplitude	reference		reference		reference		reference		reference		reference		-	

Model 4: Analysis limited to the Caucasian population.
Model 5: Excessive alcohol consumption was eliminated.
Model 6: Exclusion of participants with follow-up periods of less than 2 years.
Model 7: Additional adjustment for polygenic risk scores on top of Model 3.
Model 8: Additional adjustment for antihypertensive drugs, lipid-lowering drugs, hypoglycemic drugs, and aspirin in addition to Model 3.
Model 9: Additional adjustments for polygenic risk scores, antihypertensive drugs, lipid-lowering drugs, hypoglycemic drugs, and aspirin in addition to Model 3.
Model 10: Exclusion of nonliver-related deaths and analysis of the associations between relative amplitude and MASLD and cirrhosis using a competing risk model.

Table S12. Basic information of the cross-sectional analysis.

Characteristics	Total	Normal circadian rhythmicity (Q2-4 of relative amplitude)	Abnormal circadian rhythmicity (Q1 of relative amplitude)	P value
Sample size, n (%)	15106 (100%)	3271 (21.7%)	11835 (78.3%)	
Male, n (%)	11835 (78.3%)	1806 (55.2%)	5164 (43.6%)	<0.001
Age (years)	55.30 (7.59)	55.48 (7.77)	55.25 (7.54)	0.132
White, n (%)	14823 (98.1%)	3164 (96.7%)	11659 (98.5%)	<0.001
Townsend deprivation index	-1.93 (2.70)	-1.48 (2.94)	-2.05 (2.62)	<0.001
Season				<0.001
Spring	3144 (20.8%)	717 (21.9%)	2427 (20.5%)	
Summer	3426 (22.7%)	655 (20.0%)	2771 (23.4%)	
Autumn	4124 (27.3%)	853 (26.1%)	3271 (27.6%)	
Winter	4412 (29.2%)	1046 (32.0%)	3366 (28.4%)	
Education				0.169
College or University degree	7306 (48.4%)	1555 (47.5%)	5751 (48.6%)	
A AS level or equivalent	2011 (13.3%)	463 (14.2%)	1548 (13.1%)	
O levels or equivalent	3396 (22.5%)	712 (21.8%)	2684 (22.7%)	
Other	2393 (15.8%)	541 (16.5%)	1852 (15.6%)	
Alcohol intake (g/d)	10.82 (9.96)	11.25 (11.30)	10.70 (9.55)	0.005
Smoking status, n (%)				<0.001
Never	9291 (61.5%)	1885 (57.6%)	7406 (62.6%)	
Previous	5017 (33.2%)	1145 (35.0%)	3872 (32.7%)	
Current	798 (5.3%)	241 (7.4%)	557 (4.7%)	
BMI (kg/m^2)	26.32 (4.13)	27.89 (4.75)	25.89 (3.83)	<0.001
Physical activity (MET hours/week)	41.09 (39.42)	35.99 (37.47)	42.50 (39.83)	<0.001
Hypertension, n (%)	5912 (39.1%)	1360 (41.6%)	4552 (38.5%)	0.001
Diabetes, n (%)	342 (2.3%)	145 (4.4%)	197 (1.7%)	<0.001
HbAIC (%)	34.98 (4.95)	35.63 (6.05)	34.80 (4.59)	<0.001
Triglyceride (mmol/L)	1.62 (0.94)	1.81 (1.07)	1.57 (0.89)	<0.001
Cholesterol (mmol/L)	5.74 (1.08)	5.66 (1.13)	5.76 (1.07)	<0.001
HDL-c (mmol/L)	1.49 (0.38)	1.39 (0.37)	1.52 (0.38)	<0.001
LDL-c (mmol/L)	3.58 (0.83)	3.56 (0.86)	3.59 (0.82)	0.067

Albumin (g/L)	45.39 (2.52)	45.20 (2.54)	45.44 (2.51)	<0.001
C-reactive protein (mg/L)	2.02 (3.60)	2.48 (3.99)	1.89 (3.48)	<0.001

BMI: Body mass index; HbAIC: Glycated hemoglobin; HDL-c: High-density lipoprotein cholesterol; LDL-c: Low-density lipoprotein cholesterol

Table S13. Linear regression analysis of the associations between the relative amplitude and the logPDFF and cT1 scores.

	**Model 1**		**Model 2**		**Model 3**	
	**HR (95%Cl)**	**P**	**HR (95%Cl)**	**P**	**HR (95%Cl)**	**P**
MRI-logPDFF						
Q1 of relative amplitude	0.28(0.26,0.31)	<0.001	0.25 (0.22,0.28)	<0.001	0.10 (0.07,0.12)	<0.001
Q2-4 of relative amplitude	reference		reference		reference	
MRI-cT1 score						
Q1 of relative amplitude	19.79 (17.45,22.12)	<0.001	18.34 (16.02,20.68)	<0.001	8.35 (6.15,10.56)	<0.001
Q2-4 of relative amplitude	reference		reference		reference	

Model 1: Without adjustment for confounding factors.

Model 2: Additional adjustment for sex and age.

Model 3: Additional adjustment for race, quartile deprivation index, season, education, alcohol consumption, smoking status, body mass index, physical activity, hypertension, diabetes, triglycerides and cholesterol.

Figure S1. Cumulative hazard of the relative amplitude with CLD.

Model 1: Without adjustment for confounding factors.

Model 2: Additional adjustment for sex and age.

Model 3: Additional adjustment for race, town deprivation index, season, education, alcohol consumption, smoking status, body mass index, physical activity, hypertension, diabetes, triglycerides and cholesterol.

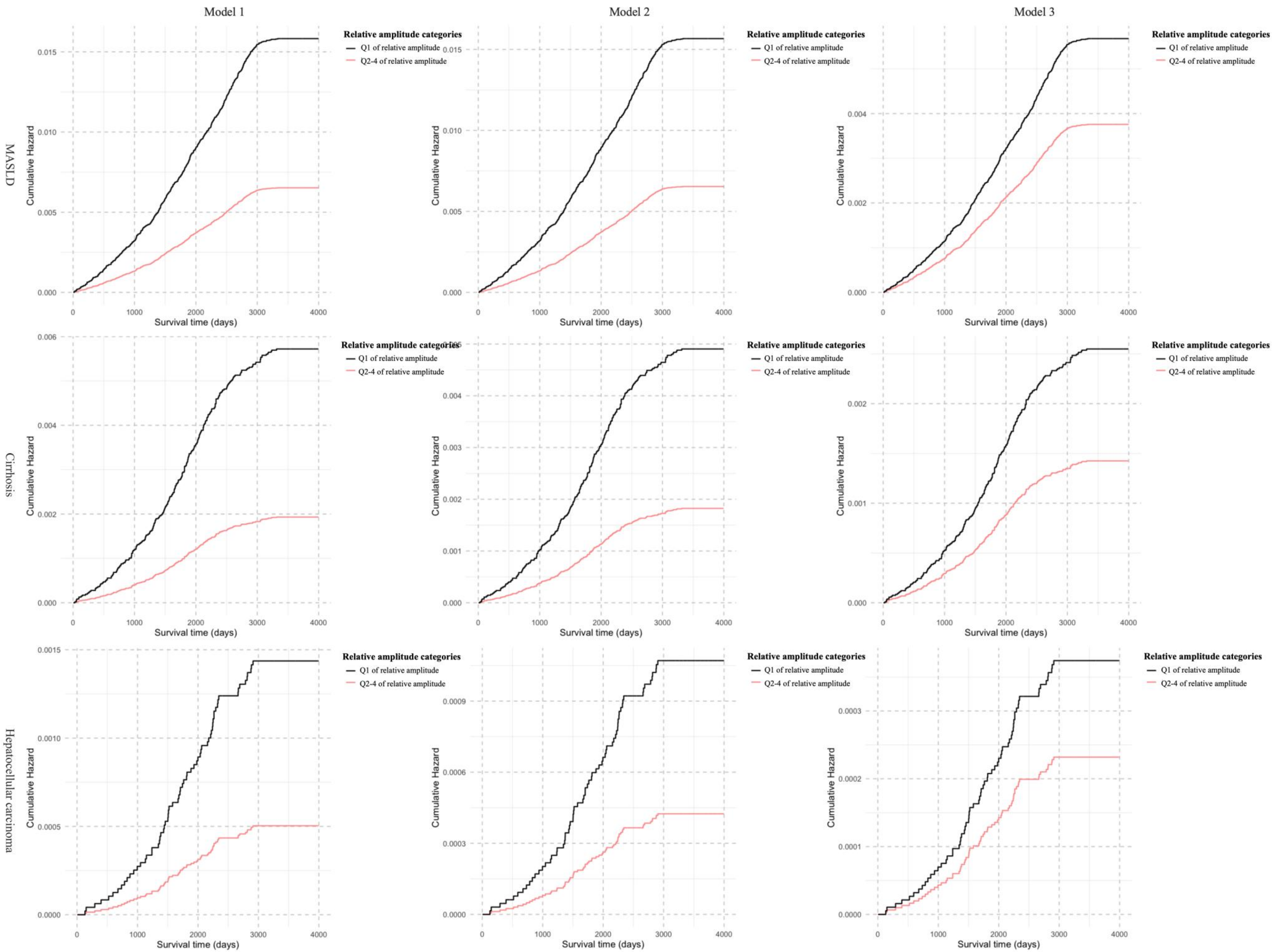

Model 1
Model 2
Model 3
MASLD
Cirrhosis
Hepatocellular carcinoma
Cumulative Hazard
Survival time (days)
Relative amplitude categories
Q1 of relative amplitude
Q2-4 of relative amplitude
